# PAR2 Regulates Cardiac Pressure and Vascular Function Through Context-Dependent Signalling Mechanisms

**DOI:** 10.3390/cimb48070727

**Published:** 2026-07-16

**Authors:** Joselia Carlos, Filip Konecny, Maryia Ryskina, John J. McGuire

**Affiliations:** 1 Department of Medical Biophysics, Schulich School of Medicine and Dentistry, Western University, London, ON N6A 5C1, Canada; joseliacarlos6@gmail.com (J.C.); filkon76@gmail.com (F.K.); mryskina@uwo.ca (M.R.); 2 Independent Researcher, Toronto, ON M2J 5C2, Canada; 3 Department of Surgery, Division of Plastic Surgery, McMaster University, Hamilton, ON L8P 3A9, Canada; 4 Comparative Medicine Core Facility, Lee Moffitt Cancer Center & Research Institute, Tampa, FL 33612, USA

**Keywords:** proteinase-activated receptor-2 (PAR2), cardiac pressure–volume analysis, vascular tone and stiffness, G protein-coupled receptor signalling, cardiovascular phenotype

## Abstract

Proteinase-activated receptor-2 (PAR2) regulates cardiac pressure and vascular function through context-dependent mechanisms that integrate G protein-coupled receptor signalling. We investigated the baseline cardiovascular phenotype of PAR2-deficient (PAR2^−/−^) mice and the acute in vivo effects of the PAR2 agonist *trans*-cinnamoyl-Leu-Ile-Gly-Arg-Leu-Orn-amide (tcLIGRLO). Left ventricular pressure–volume (PV) analysis revealed that PAR2^−/−^ mice had elevated systolic and diastolic pressures with reduced end-diastolic volumes and increased ejection fraction while maintaining stroke volume and cardiac output. Thus, PAR2 deficiency produces a pressure-dominant cardiovascular state with increased mechanical work and preserved global function. In WT mice, tcLIGRLO dose-dependently reduced cardiac pressure generation, contractility, relaxation kinetics, and stroke work, whereas these effects were absent in PAR2^−/−^ mice, indicating PAR2-dependent cardiac regulation. Despite these changes, global cardiac output remained largely preserved despite modest reductions in heart rate. In contrast, tcLIGRLO elicited strain-dependent vascular responses, including altered effective aortic elastance and reduced carotid blood flow in PAR2^−/−^ mice. Ex vivo experiments showed that tcLIGRLO-induced contraction in PAR2^−/−^ arteries required Gq-dependent signalling in this experimental context. These findings identify PAR2 as a context-dependent regulator of cardiac pressure and vascular tone, in which PAR2-dependent cardiac effects and PAR2-independent, Gq-mediated vascular responses reflect distinct but interacting signalling mechanisms.

## 1. Introduction

Proteinase-activated receptors (PARs), a family of four G protein-coupled receptors (GPCR), modulate cellular responses that are central to cardiovascular, inflammatory, and metabolic diseases. Tethered ligands, integral to the receptors, activate PAR1, PAR2, and PAR4 by binding to specific sites on each. These tethered ligands are effectively hidden until proteolytic cleavage of the PAR extracellular N-terminus initiates signalling [1]. Researchers have developed synthetic PAR-activating peptides (PAR-AP) based on these tethered ligand sequences to investigate receptor-specific signalling and pharmacological modulation, although no PAR2-targeted drugs are currently approved for clinical use [2].

PAR2-activating peptides dilate blood vessels through endothelium-dependent mechanisms, lower arterial blood pressure in rodents [3,4], and increase regional blood flow in humans [5,6,7]. In PAR2-deficient (PAR2^−/−^) mice, systolic arterial pressure and pulse pressure are modestly elevated relative to wild-type (WT) controls [8]. With ageing, PAR2 deficiency is associated with diastolic dysfunction and myocardial fibrosis while systolic function is maintained [9]. These changes have been linked to increased PAR1 activity in cardiovascular tissues [4,9,10], suggesting compensatory signalling mechanisms that may contribute to altered cardiac and vascular function. While PAR2 signalling is often associated with endothelium-dependent vasodilation, it also influences vasoconstrictor responses and vascular structure [11], highlighting a broader role in coordinating vascular tone and remodelling. However, the mechanisms that integrate PAR2-dependent and compensatory signalling pathways in vivo remain unclear.

We previously showed that the PAR2-activating peptide *trans*-cinnamoyl-Leu-Ile-Gly-Arg-Leu-Orn-amide (tcLIGRLO) induces both endothelium-dependent relaxation and vascular smooth muscle (VSM) contraction in mouse femoral arteries, with the contractile response occurring independently of PAR2 and the endothelium [12]. Few studies have examined these PAR2-independent effects, and their physiological relevance in vivo remains poorly defined [13,14,15,16]. These findings raise a key question: how do PAR2-dependent and PAR2-independent signalling pathways interact to modulate cardiovascular function under physiological conditions?

YM-254890 is a selective inhibitor of Gq/11 signalling that has been widely used to distinguish Gq-dependent receptor responses from parallel signalling pathways [17]. In vascular tissues, YM-254890 attenuates contractile responses mediated through Gq-coupled signalling pathways, supporting its use as a pharmacological probe of Gq-dependent mechanisms [18]. These observations provide a pharmacological approach to evaluate whether tcLIGRLO-induced PAR2-independent contraction [12] is mediated through Gq-dependent signalling mechanisms.

In this study, we define the cardiac phenotype of PAR2^−/−^ mice and the acute hemodynamic effects of tcLIGRLO in both WT and PAR2^−/−^ mice using left ventricular pressure–volume analysis and carotid blood flow measurements. Our previous work showed that tcLIGRLO-induced VSM contraction depends on Ca^2+^-release from intracellular stores [12], consistent with Gq-coupled receptor signalling mechanisms. Here, we further investigated this mechanism using a selective inhibitor of the Gq signalling pathway. We report that PAR2^−/−^ mice exhibit a pressure-dependent cardiac phenotype characterized by elevated ventricular pressures and reduced ventricular filling with preserved cardiac output. We further show that tcLIGRLO modulates cardiac pressure generation and vascular responses in a PAR2-dependent manner in vivo, while eliciting PAR2-independent, Gq-dependent contraction in vascular smooth muscle ex vivo. These findings define a framework to examine how PAR2 integrates cardiac and vascular responses through context-dependent signalling mechanisms.

## 2. Materials and Methods

### 2.1. Chemicals

We purchased the chemicals for the preparation of buffered physiological salts solution (PSS) and phenylephrine from BioBasic Canada Inc. (Markham, ON, Canada) and Thermo Fisher Scientific (Mississauga, ON, Canada) and YM-254890 (1 mg) from Focus Biomolecules (Plymouth Meeting, PA, USA). The amide form of the peptide tcLIGRLO was synthesized by GenScript (Piscataway, NJ, USA).

Unless otherwise stated, all drug solutions and dilutions were prepared using molecular-biology-grade deionized water. We used 25 mM HEPES buffer (pH 7.4) and dimethyl sulfoxide (DMSO) to prepare stock solutions of tcLIGRLO (10 mM) and YM-254890 (2 mM), respectively. Stock solutions were stored at −20 °C. Dilutions of stock solutions were prepared on the day of experiments. The final concentration of DMSO in experimental solutions did not exceed 0.1% (*v*/*v*) PSS.

### 2.2. Physiological Salts Solution (PSS)

For ex vivo studies, PSS (pH 7.4) comprised (in mM) 118 NaCl, 4.7 KCl, 0.87 MgSO_4_, 0.86 KH_2_PO_4_, 2.5 CaCl_2_, 10 D-glucose, 25 NaHCO_3_, and 25 HEPES and was bubbled continuously with 95% O_2_/5% CO_2_ gas.

We used ice-cooled (4 °C) PSS for temporary storage and arterial ring dissections and warmed PSS (37 °C) for functional studies.

### 2.3. Experimental Animals

We used male and female wild-type (WT; C57BL/6 background controls) and B6.Cg.F2rl1^tm1MSB1b^/J (PAR2^−/−^) mice. WT mice were obtained from Jax Laboratories (Bar Harbor, ME, USA) and Charles River Laboratories (Saint-Constant, QC, Canada). PAR2^−/−^ mice were derived from a breeding colony (stock no. 4993, Jax Laboratories) maintained at Western University.

Mice (*n* = 72 animals for in vivo experiments; *n* = 13 animals for ex vivo experiments) were 11–39 weeks of age and weighed >18 g at the time of experimentation as a condition of the approved protocols.

The Institutional Animal Care Committee approved all protocols and procedures in accordance with Canadian Council on Animal Care guidelines (protocol numbers, dates of approval: 2018-109, 1 October 2018; 2022-129, 1 January 2023; 2024-069, 1 August 2024). This study complies with ARRIVE guidelines 2.0. Animals received TekLad Laboratory Animal Diet 7913 (Inotiv, Indianapolis, IN, USA) and water ad libitum. Mice were housed in a specific pathogen-free barrier facility in ventilated cages. Environmental conditions were maintained on a 12 h light/12 h dark cycle (07:00 a.m. lights on) at controlled temperature (22–25 °C) and humidity (40–60%). All mice received from suppliers had acclimatized in the facility for >2 weeks.

For in vivo studies, animals were euthanized with isoflurane overdose followed by cardiac puncture after the measurements and experimental procedures were completed or upon reaching predefined endpoints, including unstable bradycardia, defined as sustained HR <300 bpm or loss of PV catheter signal fidelity. For ex vivo studies, mice were euthanized with cervical dislocation without anesthesia.

In vivo experiments were conducted with a single cohort of animals, and a separate group was used for ex vivo studies. We collected baseline in vivo data in all animals and tcLIGRLO treatment data in a subset of the cohort. The experimenter (F.K.) was blinded to the strains and treatments during in vivo data acquisition and analyses. For ex vivo experiments, the vessel treatment groups were randomly assigned and no blinding was applied to data acquisition and analyses. These design choices reflect the within-animal repeated-measures design and objective automated data acquisitions, which reduce operator-dependent bias. Sex was included as a biological variable in all initial analyses. Age distribution differed between sexes because the female animals weighed less and was accounted for in statistical analyses where appropriate. Experiments were conducted in one or two pairs of mice per day. Sample sizes were based on prior studies of cardiovascular phenotyping [8] and feasibility constraints; no formal power calculation was performed. No animals were excluded from analysis unless predefined criteria were met, which resulted in exclusion of three animals.

### 2.4. Surgical Procedures

On the day of experiment, we weighed and anesthetized mice using 3–4% isoflurane in oxygen (0.5 L/min total flow rate). Ophthalmic ointment was applied, and the surgical site was prepared by shaving and depilatory treatment. We used a heating pad to maintain mouse body temperature throughout each experiment.

We intubated mice and mechanically ventilated them using a volume-controlled ventilator (SAR-1000;CWE Inc., Ardmore, PA, USA) according to manufacturer guidelines adjusted for body weight to maintain physiological heart rate and oxygenation. Anesthesia was adjusted to maintain stable heart rate for most animals. We monitored and recorded anesthesia depth, physiological parameters, and rectal temperature every 10–15 min.

A calibrated transit-time flow probe (0.5 mm PS-Series, Transonic, Ithaca, NY, USA) was placed around the right carotid artery (RCA) to measure blood flow. After signal stabilization, a calibrated 1.2F pressure–volume (PV) micro-manometer catheter (Transonic-Scisense, London, ON, Canada) was inserted into the left ventricle via an apical stab incision (27–30 G needle). Signals were recorded using PowerLab A/D system (ADInstruments, Boulder, CO, USA) and LabChart Pro software (version 8).

For pharmacodynamic experiments, we administered intracardiac boluses (15 µL each) of saline (0.9% NaCl) or tcLIGRLO (0.1, 1, and 3.6 nmol/g body weight). Intracardiac administration was selected to provide rapid and reproducible delivery of tcLIGRLO to the systemic circulation during simultaneous pressure–volume and carotid blood flow measurements. This route also minimizes variability associated with venous access and pulmonary first-pass exposure during acute pharmacodynamic experiments. Doses were administered sequentially with 5–10 min washout within each animal. Doses were selected based on prior studies of acute hemodynamic responses to PAR-activating peptides [3]. Treatments were administered in a repeated-measures design within animals.

### 2.5. Pressure–Volume Calibration

Before each experiment, the PV micro-manometer catheter was immersed in isotonic saline at 35–37 °C for 20 min to equilibrate the pressure sensor prior to its calibration and use. Left ventricular volume, phase angle, and admittance magnitude channels were electronically calibrated in saline using a two-point admittance-based method implemented by the Transonic-Scisense system. Immediately before insertion into the heart, the pressure sensor was zeroed under the meniscus of a 38 °C saline solution. The system settings for blood resistivity and myocardial electrical properties were entered based on values obtained using the manufacturer’s calibration probe.

### 2.6. In Vivo Data Acquisition

Data acquisition began after stabilization of physiological parameters following surgical procedures. We recorded raw blood flow and calculated time-averaged flow. Left ventricular pressure, volume, phase angle (θ), and admittance magnitude (γ) were collected via the PV catheter. We used real-time sinusoidal tracings of θ and γ to optimize PV catheter positioning. For each mouse, a pre-baseline scan was obtained by pausing ventilation for 5 s during inspiration to reduce motion artefacts.

Hemodynamic variables were quantified as mean values over a 30–40 s interval. Baseline values of heart rate (HR), end-systolic pressure (ESP), and end-diastolic pressure (EDP) were confirmed by continuous PV monitoring.

### 2.7. PAR2^−/−^ Mouse Femoral Artery Ex Vivo Studies

Isometric contractions were measured using a Danish Myo Technology 620 Multi Wire Myograph System (Hinnerup, Denmark), as described previously [12]. Femoral arteries were excised, cleaned of connective tissue, and divided into 1–2 mm rings (2–4 per mouse). The rings were mounted with 40 µm silver-plated tungsten wires on myograph jaws between a fixed micropositioner and an isometric force transducer.

Bath chambers were filled with PSS at 37 °C and continuously aerated (95% O_2_/5% CO_2_). Vessel normalization was performed using DMT normalization software to achieve a target transmural pressure of 13.3 kPa (IC_1_/IC_100_ = 0.9). Arterial viability was assessed using KCl (30, 60, 90 mM), and tissues producing <1.5 mN force were excluded. After a ≥20 min washout, tissues were treated with vehicle or YM-254890 (0.01–0.1 µM) for 15 min prior to concentration–response curves for tcLIGRLO (0.1–50 µM) or phenylephrine (0.001–10 µM). The responses were normalized to maximal contraction induced by 90 mM K^+^. A second KCl exposure following YM-254890 treatment assessed the effects on normalization.

### 2.8. Data Analysis

We used LabChart Pro (ADInstruments, Boulder, CO, USA) and LabScribe3 (version 24; iWorx Systems, Dover, NH, USA) for acquisition of hemodynamic data and PV parameter analyses, with each data point representing a 30–40 s interval per animal. For ex vivo data, we used LabChart 8 (version 8.15) for acquisition and extraction of tension data. Data were tabulated in Microsoft Excel. Statistical analyses and nonlinear regression were performed using GraphPad Prism (version 11.0.2).

In vivo data are presented as individual values with means ± 95% confidence intervals (CIs), where *n* represents the number of animals. Ex vivo data are presented as means ± SEM.

Concentration–response data were fitted using a four-parameter logistic model:

$$Y=Bottom+\frac{Top-Bottom}{1+10^{\left((LogEC50-X)\times Hillslope\right)}}$$

Bottom was constrained to 0, and the Hill slope was shared across groups. Fit quality was assessed using R^2^ values (>0.92). Differences between curves were evaluated using extra sum-of-squares F-tests.

Baseline data were analyzed using two-way ANOVA (sex × strain). In vivo treatment effects were analyzed using mixed-effects models (sex × strain × treatment), reduced to strain × treatment when no sex effect was detected. Repeated measures were accounted for within animals. Model assumptions were assessed using residual diagnostics. Post hoc comparisons (Šídák’s test) were performed only for significant interactions. Given the age range of the animals studied, additional multiple linear regression analyses were performed to evaluate the potential contributions of age and body weight to the primary cardiovascular outcomes. Regression models included sex, strain, and sex × strain interaction terms, with age or body weight included as covariates where indicated.

Statistical significance was defined as *p* < 0.05 (*), *p* < 0.01 (**), *p* < 0.001 (***), *p* < 0.0001 (****).

## 3. Results

### 3.1. Baseline Characteristics and Cardiac Phenotype

**Figure 1:**
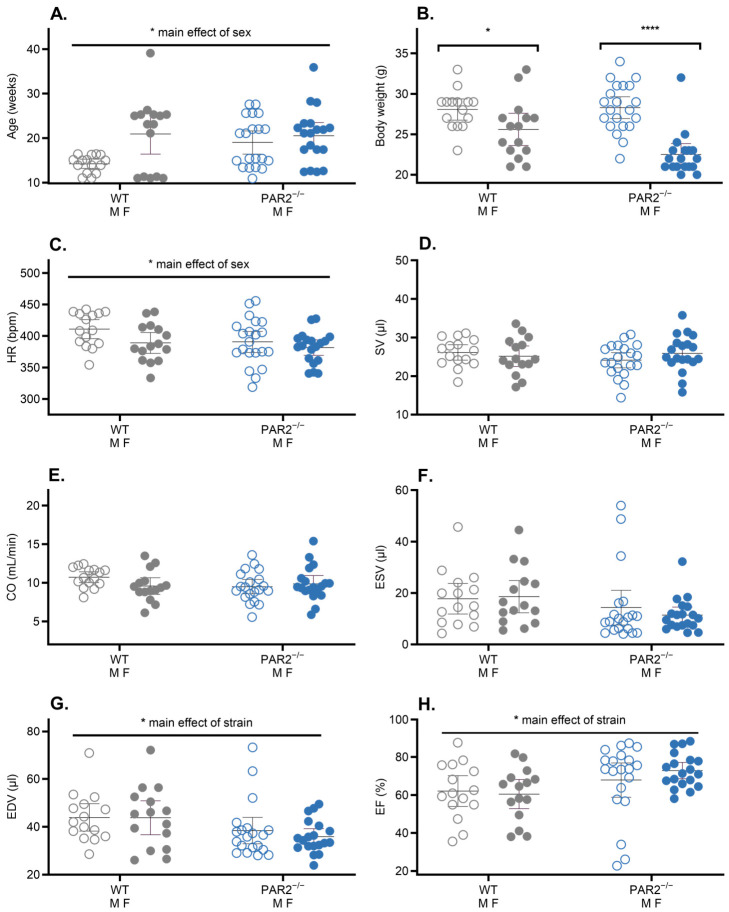
Baseline characteristics and global cardiac performance by strain and sex. Scatter plots show individual animals from four groups separated by strain and sex, with group means ± 95% CI. Wild-type control (WT) mice are shown in grey and proteinase-activated receptor-2 gene-deficient mice (PAR2^−/−^) in blue. Open circles represent males and filled circles represent females. Sample sizes were as follows: WT males (*n* = 15), WT females (*n* = 15), PAR2^−/−^ males (*n* = 20), and PAR2^−/−^ females (*n* = 19). Animals were 11–39 weeks of age. Panels show (**A**) age, (**B**) body weight, (**C**) heart rate (HR), (**D**) stroke volume (SV), (**E**) cardiac output (CO), (**F**) end-diastolic volume (EDV), (**G**) end-systolic volume (ESV), and (**H**) ejection fraction (EF). Data were analyzed using two-way ANOVA (sex × strain). Significant main effects of sex and/or strain are indicated where present. Horizontal bars denote significant ANOVA main effects. When a factor contained only two levels and no interaction was detected, and post hoc comparisons were not performed because they are equivalent to the corresponding main-effect test. Post hoc comparisons (Šídák’s multiple comparisons test) are shown only for outcomes with a significant sex × strain interaction and are indicated by brackets connecting the groups compared; non-significant differences are not annotated. Statistical significance was defined as *p* < 0.05 (*), *p* < 0.0001 (****).

To define the baseline phenotype of PAR2^−/−^ mice, we assessed cohort characteristics and global cardiac performance in male and female WT and PAR2^−/−^ animals (Figure 1).

Male mice were younger than females, whereas age did not differ significantly between strains within each sex (Figure 1A). Body weight was lower in females across strains (Figure 1B).

Despite these differences, indices of global cardiac function were largely preserved between groups. Heart rate was lower in females than males across strains (Figure 1C). Stroke volume, cardiac output, and end-systolic volume did not differ significantly between WT and PAR2^−/−^ mice or between sexes (Figure 1D–F). In contrast, end-diastolic volume was reduced in PAR2^−/−^ mice relative to WT, resulting in a corresponding increase in ejection fraction (Figure 1G,H). No consistent sex-dependent effects or sex × strain interactions were detected for these outcomes. Together, these findings indicate that PAR2^−/−^ mice exhibit reduced ventricular filling and increased ejection fraction while maintaining stroke volume and cardiac output comparable to WT controls.

**Figure 2:**
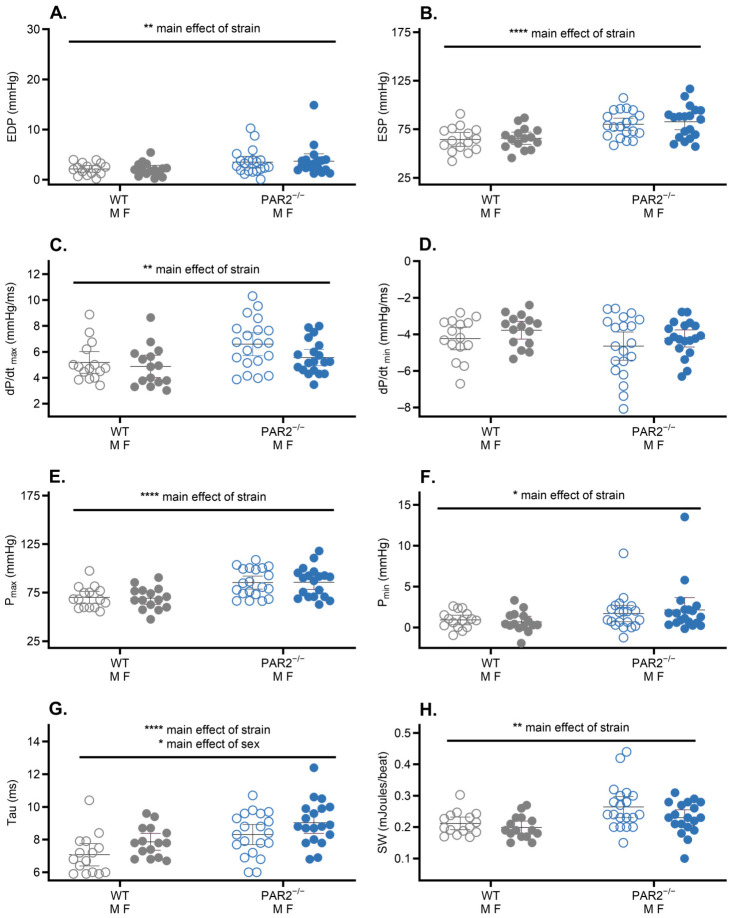
Left ventricular pressure dynamics and cardiac work by strain and sex. Scatter plots show individual animals from four groups separated by strain and sex, with group means ± 95% CI. Wild-type controls (WT) mice are shown in grey and proteinase-activated receptor-2 gene-deficient mice (PAR2^−/−^) in blue. Open circles represent males and filled circles represent females. Sample sizes were as follows: WT males (*n* = 15), WT females (*n* = 15), PAR2^−/−^ males (*n* = 20), and PAR2^−/−^ females (*n* = 19). Animals were 11–36 weeks of age. Panels show (**A**) end-diastolic pressure (EDP), (**B**) end-systolic pressure (ESP), (**C**) maximum rate of pressure rise (dP/dt_max_), (**D**) maximum rate of pressure decline (dP/dt_min_), (**E**) maximal ventricular pressure (P_max_), (**F**) minimal ventricular pressure (P_min_), (**G**) time constant of isovolumetric relaxation (τ), and (**H**) stroke work (SW). Data were analyzed using two-way ANOVA (sex × strain). Significant main effects of sex and/or strain are indicated where present; no sex × strain interactions were detected. Horizontal bars denote significant ANOVA main effects. When a factor contained only two levels and no interaction was detected, post hoc comparisons were not performed because they are equivalent to the corresponding main-effect test. Statistical significance was defined as *p* < 0.05 (*), *p* < 0.01 (**), *p* < 0.0001 (****).

To further define the haemodynamic basis of this phenotype, left ventricular pressure dynamics and indices of cardiac work were examined (Figure 2).

### 3.2. Left Ventricular Pressure Dynamics and Mechanical Work Differ by Strain

Left ventricular pressure and work indices revealed clear strain-dependent differences (Figure 2). PAR2^−/−^ mice exhibited higher pressures during both diastole and systole, including increased end-diastolic pressure, end-systolic pressure, and rates of pressure development during isovolumetric contraction; maximal and minimal ventricular pressure were also elevated, indicating enhanced systolic pressure generation (Figure 2A–C,E,F).

Indices of diastolic and energetic function were similarly altered. Rate of ventricular pressure decline during isovolumetric relaxation did not differ between strains (Figure 2D). However, the time constant of isovolumetric relaxation was increased in PAR2^−/−^ mice, consistent with impaired relaxation kinetics, and stroke work was higher relative to WT (Figure 2G,H). Together, these data describe a PAR2^−/−^ cardiac phenotype with lower ventricular filling but elevated pressures and increased mechanical work despite preserved stroke volume and cardiac output.

These findings indicate that the elevated pressures and reduced ventricular filling observed in PAR2^−/−^ mice occur in the absence of impaired global output, suggesting altered loading conditions or compensatory signalling rather than primary systolic dysfunction.

### 3.3. tcLIGRLO Produces Strain-Dependent Changes in Cardiac Pressure, Work, and Vascular Responses

**Figure 3:**
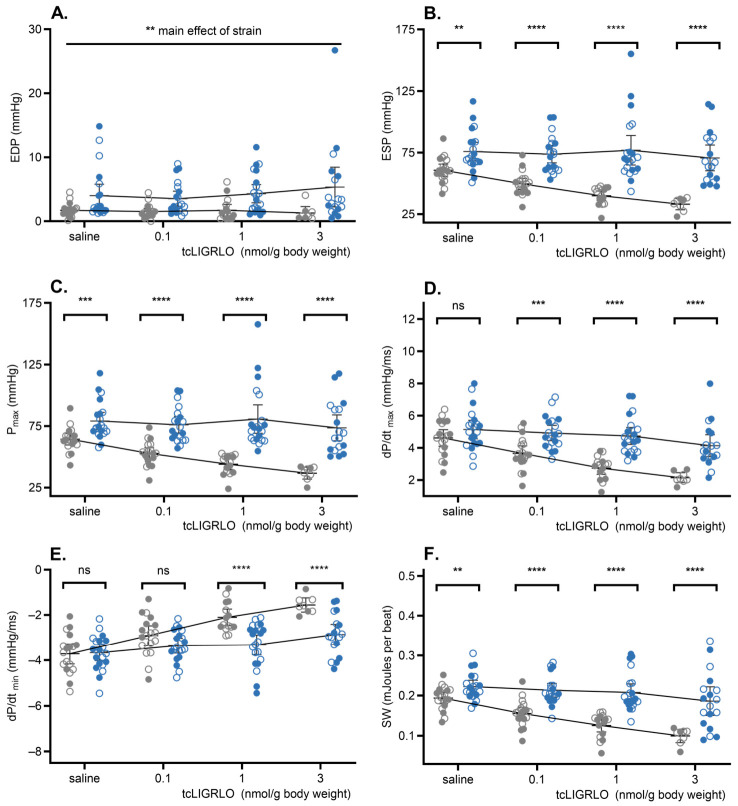
Effects of tcLIGRLO on left ventricular pressure dynamics and cardiac work by strain. Scatter plots show individual mice plotted by strain across increasing doses of *trans*-cinnamoyl-Leu-Ile-Gly-Arg-Leu-Orn-amide (tcLIGRLO), with means ± 95% CIs and light lines connecting means. Wild-type controls (WT) mice are shown in grey and proteinase-activated receptor-2 gene-deficient mice (PAR2^−/−^) in blue. Open circles represent males and filled circles represent females; sex is consolidated for visualization. Treatments were saline and tcLIGRLO (0.1, 1, and 3 nmol/g body weight). Sample sizes were: WT (*n* = 19; 10 males, 9 females) and PAR2^−/−^ (*n* = 21; 11 males, 10 females). Animals in this pharmacological subset were 11–39 weeks of age. Panels show (**A**) end-diastolic pressure (EDP), (**B**) end-systolic pressure (ESP), (**C**) maximal ventricular pressure (P_max_), (**D**) maximum rate of pressure rise (dP/dt_max_), (**E**) maximum rate of pressure decline (dP/dt_min_) and (**F**) stroke work (SW). Data were analyzed using mixed-effects models. No main effects of sex were detected; analysis was therefore reduced to strain × treatment models. Horizontal bars denote significant ANOVA main effects. When a factor contained only two levels and no interaction was detected, post hoc comparisons were not performed because they are equivalent to the corresponding main-effect test. Significant strain × treatment interactions were tested using Šídák’s post hoc comparisons between strains at each dose and are indicated by brackets connecting the groups compared; all post hoc results, including non-significant (ns) comparisons, are annotated. WT sample size was reduced at higher doses due to attainment of predefined hemodynamic endpoint criteria; mixed-effects modelling accounts for unequal group sizes. Significance thresholds were *p* < 0.01 (**), *p* < 0.001 (***), *p* < 0.0001 (****).

**Figure 4:**
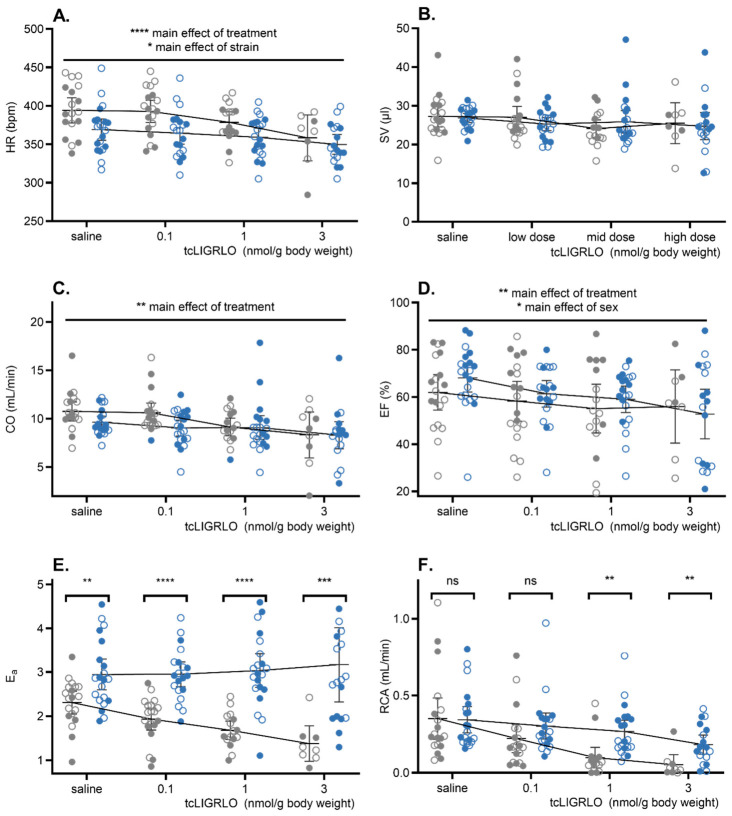
Effects of tcLIGRLO on global cardiac performance and vascular output by strain. Scatter plots show individual mice plotted by strain across increasing doses of *trans*-cinnamoyl-Leu-Ile-Gly-Arg-Leu-Orn-amide (tcLIGRLO), with means ± 95% CIs and light lines connecting means. Wild-type control (WT) mice are shown in grey and proteinase-activated receptor-2 gene-deficient mice (PAR2^−/−^) in blue. Open circles indicate males and filled circles indicate females; sex is consolidated for visualization. Treatments were saline and tcLIGRLO (0.1, 1, and 3 nmol/g body weight). Sample sizes were: WT: (*n* = 19; 10 males, 9 females) and PAR2^−/−^ (*n* = 21; 11 males, 10 females). Animals in this pharmacological subset were 11–39 weeks of age. Panels show (**A**) heart rate (HR), (**B**) stroke volume (SV), (**C**) cardiac output (CO), (**D**) ejection fraction (EF), (**E**) effective aortic elastance (E_a_, ESP/SV), and (**F**) right carotid artery (RCA) blood flow. Mixed-effects analysis revealed main effects of treatment and strain in panel (**A**) and main effects of treatment and sex in panel (**C**), with no interactions. Panels (**B**,**D**) showed no significant effects. Horizontal bars denote significant ANOVA main effects. When a factor contained only two levels and no interaction was detected, post hoc comparisons were not performed because they are equivalent to the corresponding main-effect test. Panels (**E**,**F**) exhibited strain × treatment interactions evaluated using Šídák’s post hoc tests and are denoted by brackets connecting the groups compared; all post hoc outcomes, including non-significant (ns) comparisons, are annotated. WT attrition at higher doses reflects predefined endpoint criteria and is accounted for by mixed-effects modelling. Significance was defined as *p* < 0.05 (*), *p* < 0.01 (**), *p* < 0.001 (***), *p* < 0.0001 (****).

To evaluate the in vivo cardiac and vascular effects of tcLIGRLO, saline and increasing peptide doses were administered intraventricularly while left ventricular function and right carotid blood flow were recorded simultaneously (Figure 3 and Figure 4).

tcLIGRLO had little effect on diastolic filling, as end-diastolic pressure did not change significantly in either strain (Figure 3A). In contrast, WT mice exhibited clear dose-dependent reductions in end-systolic pressure and maximal ventricular pressure, accompanied by decreases in the rates of pressure development and decline and a corresponding reduction in stroke work (Figure 3B–F). In PAR2^−/−^ mice, these responses were absent, with no significant dose-dependent changes in systolic pressure, contractility, relaxation kinetics, or stroke work across treatment levels.

Despite these marked effects on left ventricular pressure and work, the effects of tcLIGRLO on global cardiac performance were more limited (Figure 4). Heart rate, cardiac output, and ejection fraction were modestly reduced in both WT and PAR2^−/−^ mice (Figure 4A,C,D). Stroke volume was not significantly altered in either strain (Figure 4B).

In contrast, vascular load and peripheral output showed strain-dependent responses. Effective aortic elastance (E_a_) was reduced in a dose-dependent manner in WT mice but not in PAR2^−/−^ mice. Right carotid artery blood flow also decreased with increasing tcLIGRLO dose in both strains, but responses in PAR2^−/−^ mice were rightward shifted relative to WT, indicating reduced sensitivity.

Together, these findings demonstrate that tcLIGRLO suppresses left ventricular pressure generation and cardiac work in a PAR2-dependent manner, while global cardiac output remains largely preserved. These cardiac effects are accompanied by strain-dependent changes in afterload and peripheral blood flow, indicating coordinated but distinct regulation of cardiac and vascular responses.

### 3.4. Gq Inhibition Abolishes tcLIGRLO-Induced Contraction in PAR2^−/−^ Femoral Arteries

**Figure 5:**
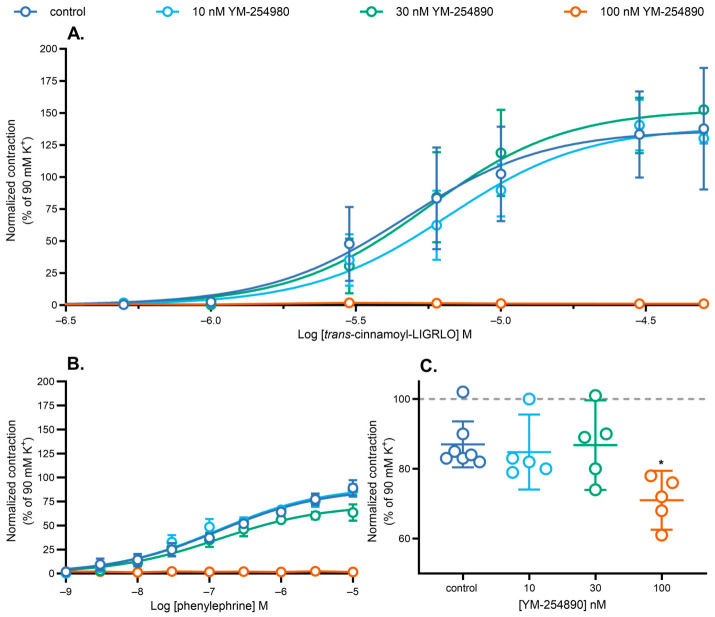
Effects of Gq inhibition on agonist-induced contraction in PAR2^−/−^ femoral arteries. Concentration–response and functional data were obtained from isolated femoral arteries of PAR2^−/−^ mice, treated ex vivo with YM-254890, a selective Gq inhibitor. Panels show (**A**) *trans*-cinnamoyl-Leu-Ile-Gly-Arg-Leu-Orn-amide (tcLIGRLO), (**B**) phenylephrine concentration–response curves, and (**C**) repeated contractions to 90 mM K^+^ in control (0.1% DMSO (*v*/*v*) physiological salts solution) and YM-254890 (10, 30, 100 nM) treatment groups, expressed as normalized contraction (% of 90 mM K^+^). Data are shown as means ± SEM ((**A**): *n* = 4 males; (**B**): *n* = 5–6 both sexes) or individual values with means ± 95% CIs ((**C**): *n* = 5–7 males). Animals were 18–35 weeks of age. The 100 nM groups did not conform to a sigmoidal fit and are shown without curve fitting. Data in (**C**) were analyzed using one-way ANOVA with a significant effect of treatment followed by Šídák’s multiple comparisons test vs control; the 100 nM group differed from control. Non-significant comparisons are not annotated. Statistical significance was defined as *p* < 0.05 (*).

To determine whether the tcLIGRLO-induced strain-dependent vascular responses observed in vivo are mediated by Gq-dependent signalling, we examined the effects of YM-254890 on tcLIGRLO-induced contraction in isolated femoral arteries from PAR2^−/−^ mice (Figure 5).

Under control conditions, tcLIGRLO elicited a robust concentration-dependent increase in normalized contraction (Figure 5A). Treatment with 10 and 30 nM YM-254890 produced minimal effects, whereas 100 nM abolished tcLIGRLO-induced contraction across all concentrations, resulting in loss of a sigmoidal dose–response relationship. A similar pattern was observed with phenylephrine, where contractions were progressively attenuated by YM-254890, with near-complete inhibition at 100 nM (Figure 5B).

To assess whether inhibition extended beyond receptor-mediated mechanisms, contractions to 90 mM K^+^ were examined (Figure 5C). YM-254890 at 10 and 30 nM did not alter K^+^-induced contraction, whereas 100 nM significantly reduced responses relative to control.

Together, these findings identify a Gq-dependent mechanism underlying agonist-induced contraction in PAR2^−/−^ arteries, providing a mechanistic framework to interpret the strain-dependent cardiac and vascular responses to tcLIGRLO observed in vivo.

## 4. Discussion

In this study, we define the cardiac phenotype of PAR2^−/−^ mice and determine the acute in vivo effects of tcLIGRLO (a PAR2 agonist with PAR2-independent effects in this context) on cardiac and vascular function. PAR2^−/−^ mice exhibit elevated left ventricular systolic and diastolic pressures with reduced end-diastolic volumes, resulting in increased ejection fraction while maintaining stroke volume and cardiac output. These baseline differences establish a pressure-dominant cardiac phenotype characterized by increased mechanical work. tcLIGRLO produces dose-dependent reductions in left ventricular pressure generation and cardiac work in WT mice, whereas these tcLIGRLO-induced effects are absent in PAR2^−/−^ mice, indicating a PAR2-dependent mechanism. Despite marked tcLIGRLO-induced changes in pressure and work, global cardiac output remains largely preserved despite modest reductions in heart rate, while vascular load and peripheral blood flow exhibit strain-dependent responses. tcLIGRLO-induced contraction in PAR2^−/−^ arteries is mediated predominantly by Gq-dependent signalling, providing a mechanistic framework to interpret the PAR2-independent vascular effects of tcLIGRLO observed in vivo. In this context, ‘context-dependent’ refers to the differential engagement of PAR2-dependent and PAR2-independent signalling across experimental conditions, including intact cardiovascular function in vivo and isolated VSM responses ex vivo.

The cardiac and vascular phenotype observed here is consistent with prior work demonstrating that PAR2 deficiency produces modest increases in systolic and pulse pressures without changes to diastolic pressure [4,8] while maintaining global cardiac output under basal conditions [9]. Our findings extend earlier observations by showing that pressure-dependent changes in ventricular function occur at an earlier stage, without loss of cardiac output. This profile aligns with studies in which systolic performance remains preserved in PAR2^−/−^ mice, whereas diastolic dysfunction and fibrosis develop with ageing [9]. The elevated pressures and impaired relaxation observed here are consistent with progression toward age-dependent diastolic dysfunction. Whether these pressure changes reflect primary cardiac adaptations or altered loading conditions remains to be determined. No consistent sex-dependent effects were detected, indicating that the observed phenotype of PAR2^−/−^ is primarily strain-dependent under these experimental conditions.

At the vascular level, PAR2 signalling regulates tone through endothelium-dependent mechanisms [3,4,12,19] while separate studies demonstrate roles in inflammatory and structural pathways [11,20,21,22]. The strain-dependent responses to tcLIGRLO (which also exhibits PAR2-independent effects in this context) support a role for PAR2 in coordinating cardiac and vascular function, where loss of PAR2 alters tcLIGRLO-induced pressure modulation and vascular responsiveness. Effective aortic elastance (E_a_) integrates ventricular pressure generation and stroke volume and is commonly used as an index of arterial load imposed on the left ventricle. The reduction in E_a_ observed in WT mice following tcLIGRLO administration is consistent with a decrease in the arterial load against which the ventricle ejects blood. In contrast, the absence of a comparable reduction in PAR2^−/−^ mice indicates an altered vascular response to tcLIGRLO and is consistent with the strain-dependent differences observed in carotid blood flow. Together, these findings support a role for PAR2 in coordinating cardiovascular responses that influence both ventricular pressure generation and vascular load. The Gq-dependent mechanism identified in PAR2^−/−^ arteries indicates that PAR2-independent vasoconstrictor pathways remain intact and may be enhanced in the absence of PAR2 signalling in this experimental context. This interpretation is consistent with evidence that PAR2 deficiency increases vasoconstrictor response and contributes to vascular remodelling and stiffness [11] without impairing endothelial vasodilation [10]. These findings support a hierarchical model in which loss of PAR2 signalling shifts receptor balance toward PAR1-mediated pathways, with downstream convergence on Gq-dependent signalling mechanisms that govern vascular contractile responses.

The divergence between PAR2-dependent and PAR2-independent responses likely reflects compensatory and parallel signalling pathways within the PAR receptor system. PAR2 deficiency is associated with increased PAR1 activity in cardiovascular tissues [4,10], which has been linked to downstream pro-fibrotic signalling and altered cardiac function [9]. Elevated pressures and increased mechanical work observed in PAR2^−/−^ mice are consistent with a shift toward PAR1-mediated signalling, while preserved endothelial function indicates that loss of PAR2 alters the balance between opposing pathways rather than uniformly impairing vascular relaxation. The Gq-dependent inhibition of contraction further suggests that vasoconstrictor responses converge on shared downstream signalling networks in PAR2^−/−^. In this framework, PAR2 functions to modulate Gq-coupled signalling, and its absence unmasks or amplifies these pathways.

These findings have important implications for understanding PAR2 as a therapeutic target. Inhibition of PAR2 may reduce inflammatory signalling and vascular injury [20,21] but may also promote pro-fibrotic pathways and structural remodelling [11]. This duality reflects observations across disease models in which PAR2 deficiency attenuates vascular inflammation while enhancing fibrosis in cardiovascular tissues [9,22]. The preservation of cardiac output despite increased mechanical load highlights a dissociation between functional compensation and underlying structural stress, which may predispose to long-term dysfunction. These observations support a strategy in which PAR2-targeted interventions are considered within the broader context of receptor cross-talk and signalling integration.

PAR2 therefore functions as a context-dependent regulator of cardiovascular physiology, integrating cardiac pressure generation, vascular tone, and structural remodelling. Loss of PAR2 signalling produces a pressure-dependent phenotype with preserved global output but altered mechanical load, while shifting vascular responses toward increased vasoconstrictor sensitivity and reduced compliance. This balance between preserved function and structural stress provides a mechanistic basis for the divergent effects of PAR2 deficiency across physiological and pathological conditions [23].

One limitation of the present study is the use of a constitutive PAR2^−/−^ mouse model. Developmental adaptations and compensatory changes in receptor signalling pathways [7,8,9] may contribute to the cardiovascular phenotype observed in adult animals and cannot be distinguished from the direct effects of PAR2 deficiency. The acute nature of the pharmacological interventions represents a second limitation, as the effects of tcLIGRLO were assessed during short-term exposure and therefore do not address longer-term cardiovascular adaptations to sustained PAR2 activation or inhibition. The route of peptide administration should also be considered when interpreting the pharmacological findings. TcLIGRLO was administered through intracardiac injection to achieve rapid and reproducible systemic delivery during simultaneous pressure–volume and carotid blood flow measurements. Although this approach facilitated acute pharmacodynamic assessment, the magnitude and time course of the responses may differ from those obtained following intravenous or other clinically relevant routes of administration. Animals spanned a range of ages, and male groups were modestly younger than female groups. However, additional regression analyses did not identify age or body weight as significant predictors of the primary cardiovascular outcomes, suggesting that age-related variation is unlikely to explain either the baseline phenotype or the pharmacological responses observed in this study. Finally, the present findings were obtained in mice and were not designed to assess long-term cardiovascular outcomes. Future studies employing longitudinal approaches, inducible or tissue-specific models of PAR2 deficiency, and clinically relevant disease models will be required to further define the translational significance of PAR2-dependent and PAR2-independent signalling mechanisms in cardiovascular physiology.

## 5. Conclusions

PAR2 deficiency reveals a context-dependent role in cardiovascular regulation. Loss of PAR2 signalling produces a pressure-dominant cardiac phenotype characterized by elevated ventricular pressures, reduced ventricular filling, and increased mechanical work, while preserving global cardiac output. These findings highlight a divergence between maintained cardiac performance and increased mechanical load.

At the vascular level, PAR2 deficiency increases vasoconstrictor sensitivity and reduces compliance while preserving endothelial vasodilator function. These changes are accompanied by alterations in vascular load and peripheral blood flow that reflect disrupted integration of cardiac and vascular responses. The identification of Gq-dependent signalling as a dominant mechanism for agonist-induced contraction indicates that PAR2 modulates shared signalling pathways rather than acting as an isolated regulator.

PAR2 functions to balance pathways that regulate vascular tone, inflammation, and structural remodelling. Loss of PAR2 preserves aspects of cardiac performance while promoting conditions that favour increased stiffness and fibrosis. These context-dependent effects underscore the importance of considering both hemodynamic regulation and structural remodelling in the development of PAR2-targeted therapeutic strategies [23].

## Data Availability

All data for this study are contained within the article and any additional data sharing will be considered by the corresponding author upon request.

## Abbreviations

The following abbreviations are used in this manuscript:

bpm	beats per minute
Ca^2+^	calcium ion
CO	cardiac output
dP/dt_max_	maximum rate of pressure rise
dP/dt_min_	maximum rate of pressure decline
E_a_	effective aortic elastance
EC_50_	half-maximal effective concentration
EDP	end-diastolic pressure
EDV	end-diastolic volume
EF	ejection fraction
ESP	end-systolic pressure
ESV	end-systolic volume
GPCR	G protein-coupled receptor
HEPES	4-(2-hydroxyethyl)-1-piperazineethanesulfonic acid
HR	heart rate
IC_1_/IC_100_	internal circumference ratio used for vessel normalization
K^+^	potassium ion
ns	non-significant
PAR	proteinase-activated receptor
PAR-AP	PAR-activating peptide
PAR1	proteinase-activated receptor-1
PAR2	proteinase-activated receptor-2
PAR4	proteinase-activated receptor-4
PAR2^−/−^	PAR2-deficient
P_max_	maximal ventricular pressure
P_min_	minimal ventricular pressure
PSS	physiological salts solution
PV	pressure–volume
RCA	right carotid artery
SV	stroke volume
SW	stroke work
tcLIGRLO	*trans*-cinnamoyl-Leu-Ile-Gly-Arg-Leu-Orn-amide
VSM	vascular smooth muscle
WT	wild-type
YM-254890	selective Gq inhibitor
τ	time constant of isovolumetric relaxation
